# Obesity programmed by prenatal dexamethasone and postnatal high-fat diet leads to distinct alterations in nutrition sensory signals and circadian-clock genes in visceral adipose tissue

**DOI:** 10.1186/s12944-019-0963-1

**Published:** 2019-01-18

**Authors:** Ching-Chou Tsai, Mao-Meng Tiao, Jiunn-Ming Sheen, Li-Tung Huang, You-Lin Tain, I-Chun Lin, Yu-Ju Lin, Yun-Ju Lai, Chih-Cheng Chen, Kow-Aung Chang, Hong-Ren Yu

**Affiliations:** 1 Department of Obstetrics and Gynecology, Kaohsiung Chang Gung Memorial Hospital, Chang Gung University, College of Medicine, Kaohsiung, 83301 Taiwan; 2 Graduate Institute of Clinical Medicine, Kaohsiung Medical University, Kaohsiung, 80708 Taiwan; 3 Department of Pediatrics, Kaohsiung Chang Gung Memorial Hospital, Chang Gung University, College of Medicine, 123 Ta-Pei Road, Niao Sung, Kaohsiung, 83301 Taiwan, Republic of China; 4 Department of Anesthesiology, Kaohsiung Chang Gung Memorial Hospital, Chang Gung University, College of Medicine, Kaohsiung, 83301 Taiwan

**Keywords:** Prenatal dexamethasone, Postnatal high-fat diet, Adipose tissue, Nutrition sensory signals, Circadian-clock

## Abstract

**Background:**

Prenatal dexamethasone treatment has been shown to enhance the susceptibility of offspring to postnatal high-fat (HF) diet-induced programmed obesity. We investigated the metabolic phenotypes, nutrient-sensing signal and circadian-clock genes in adipose tissue that are programmed by prenatal dexamethasone exposure and postnatal HF diet.

**Methods:**

Male offspring of Sprague-Dawley rats were divided into four experimental groups: normal diet, prenatal dexamethasone exposure, postnatal HF diet, and prenatal dexamethasone plus postnatal HF diet. Postnatal HF diet was prescribed from weaning to 6 months of age.

**Results:**

Prenatal dexamethasone and postnatal HF diet exerted synergistic effects on body weight and visceral adiposity, whereas prenatal dexamethasone and postnatal HF diet altered the metabolic profile and caused leptin dysregulation. Prenatal dexamethasone and postnatal HF diet distinctly influenced nutrient-sensing molecules and circadian-clock genes in adipose tissue. The mRNA expression of mTOR, AMPK-α2, PPAR-α, and PPAR-γ was suppressed by prenatal dexamethasone but enhanced by postnatal HF diet.

**Conclusion:**

Prenatal dexamethasone and postnatal HF treatment cause dysregulation of nutrient-sensing molecules and circadian-clock genes in visceral adipose tissue. Characterizing altered nutrient-sensing molecules and circadian-clock genes has potential therapeutic relevance with respect to the pathogenesis and treatment of prenatal stress and postnatal HF diet-related metabolic disorders.

## Background

Obesity is an emerging health problem in the world. Because population genetic variability does not change rapidly, changes in lifestyle are likely responsible for this increase. Changes in lifestyle include high-fat (HF) diet, decreased physical activity, reduced total daily period of sleep, increased exposure to bright light during the night, and nocturnal feeding [1]. Recent studies have shown the relationship between prenatal insult during fetal development and amplified risk of chronic diseases throughout life, such as obesity and other metabolic disorders [2]. Maternal obesity and excessive gestational weight gain also have been identified as factors contributing to increased obesity in offspring [3].

During human pregnancy, glucocorticoid (GC) treatment is often prescribed when preterm delivery is expected [4]. However, overexposure to GC has been observed in prenatal stress, which can lead to developmental programming of metabolic syndrome and hypertension in later life [5–7]. It has been suggested that owing to excessive fetal exposure to maternal GC, offspring of stressed rat dams were more susceptible to HF diet-induced obesity [8]. Our previous studies also supported this observation. We found that prenatal dexamethasone treatment enhances the susceptibility of offspring to postnatal HF diet-induced programmed obesity, insulin dysregulation, and hypertension [6, 9, 10].

Excess of adipose tissue is a characteristic of obesity. Emerging evidence shows adipose tissue as an active metabolic tissue that produces many adipokines with important physiological functions [11]. Leptin has been found to have an important role in metabolism regulation by stimulating energy expenditure, decreasing appetite, and improving glucose homeostasis. Leptin resistance often develops in obesity condition, which limits its biological effect [12]. In contrast, adiponectin can improve insulin sensitivity, enhance fatty acid oxidation, and energy expenditure [12], and its secretion is often decreased in obesity condition.

The ability to sense and respond to fluctuation in the levels of environmental nutrients is a necessity in life. Nutrient scarcity is a selective stress that has shaped the evolution of most cellular processes. Through nutrient-sensing cascades, cells integrate and coordinate intracellular and extracellular nutrition. In food-rich condition, the nutrient-sensing pathway tends to favor anabolism and nutrient storage [13]. Scant nutrient triggers catabolic processes, such as autophagy, to maintain homeostasis. Nutrient-sensing pathways are often dysregulated in humans with metabolic diseases [13].

The molecular connecting between circadian rhythms and metabolism have been disclosed recently [14–16]. One of the most attractive aspects is the interplay between nutrient sensing pathways and the circadian clock. The circadian system adapts to environmental changes to optimize physiological response. In addition to the central clock mastered by suprachiasmatic nuclei of the anterior hypothalamus and synchronized to the 24-h light/dark cycle, there are peripheral clocks throughout body tissues [15]. In contrast to central clock, the feeding/fasting cycles are the primary intents for clocks of peripheral tissues. Peripheral tissue clocks are sensitive to the composition and timing of food consumed. Dysregulation of circadian rhythm and inappropriate nutrition are closely related and are thought to contribute to the development of certain chronic diseases [15].

Currently, adipose tissue is also proposed to be an important target of developmental programming [17]. The dysfunction of adipose tissue is closely linked to obesity and its related disorders. Therefore, understanding adipose tissue pathology is of great importance in the identification of potential therapeutic targets for preventing and treating obesity-related disorders. The present study aimed to investigate the metabolic phenotypes, nutrient-sensing signal and circadian-clock genes in adipose tissue that are programmed by prenatal dexamethasone exposure and postnatal HF diet.

## Materials and methods

### Animals

Virgin Sprague-Dawley (SD) rats (12 to 16 weeks old) were obtained from BioLASCO Taiwan Co., Ltd. (Taipei, Taiwan), and then housed and maintained in a facility certified by the Association for the Assessment and Accreditation of Laboratory Animal Care International. The study protocol was described previously [6]. In brief, virgin female SD rats were allowed to mate with male rats for 24 h, and then separated from the male rats and housed individually. Pregnant female rats were randomly assigned to two groups after pregnancy: the vehicle and dexamethasone exposure groups. Animals in the dexamethasone group were injected intraperitoneally with 0.1 mg/kg/day dexamethasone from the gestational age of 14 to 20 days. The vehicle group received daily intraperitoneal injections of normal saline. After birth, the offspring were allowed to stay with the mother until weaning. Pups were weaned at 21 days after birth and placed in cages in groups of three until they reached 6 months of age. Male offspring were selected and divided into four groups: vehicle group (VEH), prenatal dexamethasone exposure (DEX), postnatal HF diet (VHF), and prenatal dexamethasone exposure with postnatal HF diet (DHF) (*n* = 8 for each group). VEH and DEX groups received control diet (protein 23.5%, fat 4.5%, crude fiber 5.0%, crude ash 7.0%, and water 13%; Fwusow Taiwan Co. Ltd., Taichung, Taiwan) after weaning. VHF and DHF groups received HF diet (58% high-fat diet; Research Diet, D12331) from weaning to 6 months of age. This study was performed according to the Guide for the Care and Use of Laboratory Animals of the National Institutes of Health and was approved by the Institutional Animal Care and Use Committee of Chang Gung Memorial Hospital Kaohsiung Medical Center.

### Body weight and blood pressure measurements

Body weight (BW) of the offspring was measured every month until they reached 6 months of age. Blood pressure was measured in conscious, 6-month-old rats using the indirect tail-cuff method (BP-2000; Visitech Systems, Inc., Apex, NC) [6, 18].

### Intraperitoneal glucose tolerance test

For intraperitoneal glucose tolerance test, blood samples were collected at five time-points after 8-h of fasting: before and 15, 30, 60, and 120 min after intraperitoneal injection of glucose (2 g/kg). Plasma glucose levels were determined using the enzymatic (hexokinase) method [19].

### Experimental procedures and specimen collection

Rat offspring were sacrificed at 6 months of age using xylazine and ketamine [6]. Heparinized blood samples were collected during euthanasia. Retroperitoneal fat was collected and used for further studies [6].

### Determination of plasma metabolic parameters

Plasma metabolic parameters, including the levels of triglyceride, Cholesterol, aspartate transaminase (AST), alanine transaminase (ALT), Alkaline phosphatase (ALKP), vascular endothelial growth factor (VEGF), tumor necrosis factor-α (TNF-α)(R&D Systems, Minneapolis, MN), and leptin (Biovendor RD291001200R, Brno, Czech Republic) were determined by enzyme-linked immunosorbent assays according to the manufacturers’ instructions.

### Western blotting

Retroperitoneal adipose tissue sample (50 mg) was homogenized with 500 μl PRO-PREP Protein Extraction Solution (#17081, iNtRon biotechnology, Korea). The cells were lysed by incubation on ice for 30 min, and then centrifuged at 14,000×*g* for 20 min at 4 °C. Protein concentrations were determined by using a Bio-Rad Protein Assay kit (Bio-Rad, Hercules, CA). Protein samples (100 μg) were boiled with gel-loading buffer for 5 min, subjected to 10% SDS-PAGE, and then transferred to a polyvinylidene fluoride membrane (Roche Applied Sciences, Basel, Switzerland). The membrane was blocked with PBS-Tween containing 5% skim milk, and then incubated for 2 h with the anti-rat antibody SIRT-1 (#ab110304, Abcam, Cambridge, MA) or GAPDH (Santa Cruz) diluted 1:200 in TBS containing 1% skim milk. Next, the membranes were washed five times with 0.1% T-TBS, incubated for 1 h with a peroxidase-labeled secondary antibody diluted 1:1000 in T-TBS, rinsed with T-TBS, and then developed using Chemi Doc (Bio-rad Image Lab 5.0).

### Quantitative reverse transcription-polymerase chain reaction (qRT-PCR)

qRT-PCR was performed as previously described [6]. In brief, 5 μg of extracted RNA sample was reverse-transcribed with Moloney murine leukemia virus reverse transcriptase. PCR was performed in a total reaction volume of 20 μl containing 2 μl of 1:10 diluted cDNA obtained from reverse transcribed RNA, specific primers, 2.5 mM MgCl_2,_ and Maxima SYBR Green/Fluorescein qPCR Master Mix (2X) (#K0242; Thermo Scientific, CA). The cycling protocol comprised one cycle of 10 min at 95 °C followed by 45 cycles of denaturation for 10 s at 95 °C, annealing for 20 s at 55 °C, and extension for 20 s at 72 °C. The primers used are presented in Supplementary Table 1. Each PCR primer set used for qRT-PCR was located in different exons. Serial dilutions of the standard cDNA were also used for parallel amplifications. Threshold cycles (Ct) were calculated using the Roche software (LCS480 1.5.0.39). Standard curves were plotted with Ct-versus-log cDNA quantities, and samples quantities were determined using the standard curves. For relative quantification of mRNA expression, the comparative Ct method was employed. The averaged Ct was subtracted from the corresponding averaged PPIB value of each sample, resulting in △Ct. △△Ct was obtained by subtracting the average control △Ct value from the average experimental △Ct value. The fold increase was established by calculating 2-△△Ct for the experimental versus control samples [6].

### Statistical analyses

The effects of prenatal dexamethasone and postnatal HF diet in 6-month-old rat offspring were evaluated by two-way ANOVA, with Tukey’s *post-hoc* test if the interaction was significant in the first four groups. In all tests, *P* < 0.05 was considered significant. Statistical analyses were performed using the SPSS version 20.0 software (SPSS, Inc., Chicago, IL).

## Results

### Prenatal dexamethasone and postnatal HF diet lead to different metabolic manifestations

As shown in Table 1, BW was significantly higher in the DHF group than in the VEH, DEX, and VHF groups. Using two-way ANOVA, we showed that prenatal dexamethasone treatment (Hit 1) and postnatal HF diet (Hit 2) significantly increased BW. Moreover, both treatments showed a significant interactive effect on BW (*P* = 0.048). Retroperitoneal fat weight was also significantly heavier in the DHF group than in the VEH, DEX, and VHF groups (*p* < 0.05). Comparison between groups showed a significant effect for prenatal dexamethasone treatment (Hit 1, *P* = 0.012) and postnatal HF diet (Hit 2, *p* < 0.001) with a positive interactive effect of Hit 1 and Hit 2 (*p* = 0.007). Subcutaneous fat weight was higher in the DHF group than in the other three groups, but the difference was not significant. The result of comparison between groups showed no significant effect for Hit 1 but had positive effect on Hit 2 (*p* < 0.001). Hit 1 and Hit 2 showed no interactive effect on subcutaneous fat weight gain. The changes of epididymal fat and mesenteric fat weight modulated by prenatal dexamethasone and postnatal HF diet were similar to subcutaneous fat.

**Table 1:** Body weight/ adipose tissue weight, biochemistry and other biomarkers

	VEH	DEX	VHF	DHF	H1	H2	H1 × H2
Weight (g)	666.33 ± 18.70	668.00 ± 29.50	763.33 ± 45.42	942.17 ± 61.84^*#†^	*p* = 0.045	*p* < 0.001	*p* = 0.048
Retroperitoneal fat (g)	13.96 ± 1.77	13.17 ± 1.36	28.95 ± 4.53^*^	48.50 ± 4.47^*#†^	*p* = 0.012	*p* < 0.001	*p* = 0.007
Subcutaneous fat (g)	26.78 ± 2.65	30.72 ± 3.27	56.42 ± 7.36	79.25 ± 9.91	NS	*p* < 0.001	NS
Mesenteric fat (g)	5.76 ± 0.93	5.58 ± 0.68	9.17 ± 1.54	13.91 ± 2.12	NS	*p* = 0.001	NS
Epididymal fat (g)	7.35 ± 0.41	8.05 ± 0.17	18.05 ± 1.46	21.42 ± 1.65	NS	*p* < 0.001	NS
AST (U/L)	128.25 ± 45.53	97.40 ± 9.37	146.33 ± 11.32	248.50 ± 28.92^#†^	NS	*p* = 0.004	*p* = 0.018
ALT (U/L)	85.75 ± 52.12	44.80 ± 2.46	78.17 ± 11.58	156.67 ± 9.65^#†^	NS	*p* = 0.026	*p* = 0.012
T-Cholesterol (mg/dL)	88.50 ± 10.74	107.40 ± 17.72	81.17 ± 5.34	63.50 ± 5.49	NS	*p* = 0.025	NS
Triglyceride (mg/dL)	104.75 ± 5.20	136.60 ± 30.76	54.00 ± 6.67	57.67 ± 8.64	NS	*p* = 0.001	NS
ALK-P (U/L)	65.50 ± 11.30	55.80 ± 3.73	49.00 ± 4.68	62.67 ± 7.54	NS	NS	NS
Leptin (ng/ml)	2.66 ± 0.30	2.58 ± 0.99	4.62 ± 0.48	4.39 ± 0.33	NS	*p* < 0.001	NS
TNFα (pg/ml)	7.81 ± 0.48	8.68 ± 0.18	11.29 ± 0.14	11.36 ± 0.54	NS	*p* < 0.001	NS
VEGF (pg/ml)	15.21 ± 0.24	15.51 ± 0.29	16.53 ± 0.23	16.47 ± 0.28	NS	*p* < 0.001	NS

*AST* aspartate transaminase, *ALT* alanine aminotransferase, *ALK-P* Alkaline phosphatase, *VEGF* vascular endothelial growth factor, *VEH* prenatal saline vehicle /postnatal normal diet group, *DEX* prenatal dexamethasone exposure /postnatal normal diet group, *VHF* prenatal saline vehicle /postnatal high fat diet group, *DHF* prenatal dexamethasone exposure /postnatal high-fat diet group. * as compared with VEH, *p* < 0.05; # as compared with DEX, *p* < 0.05; † as compared with VHF, *p* < 0.05

**Fig. 1:**
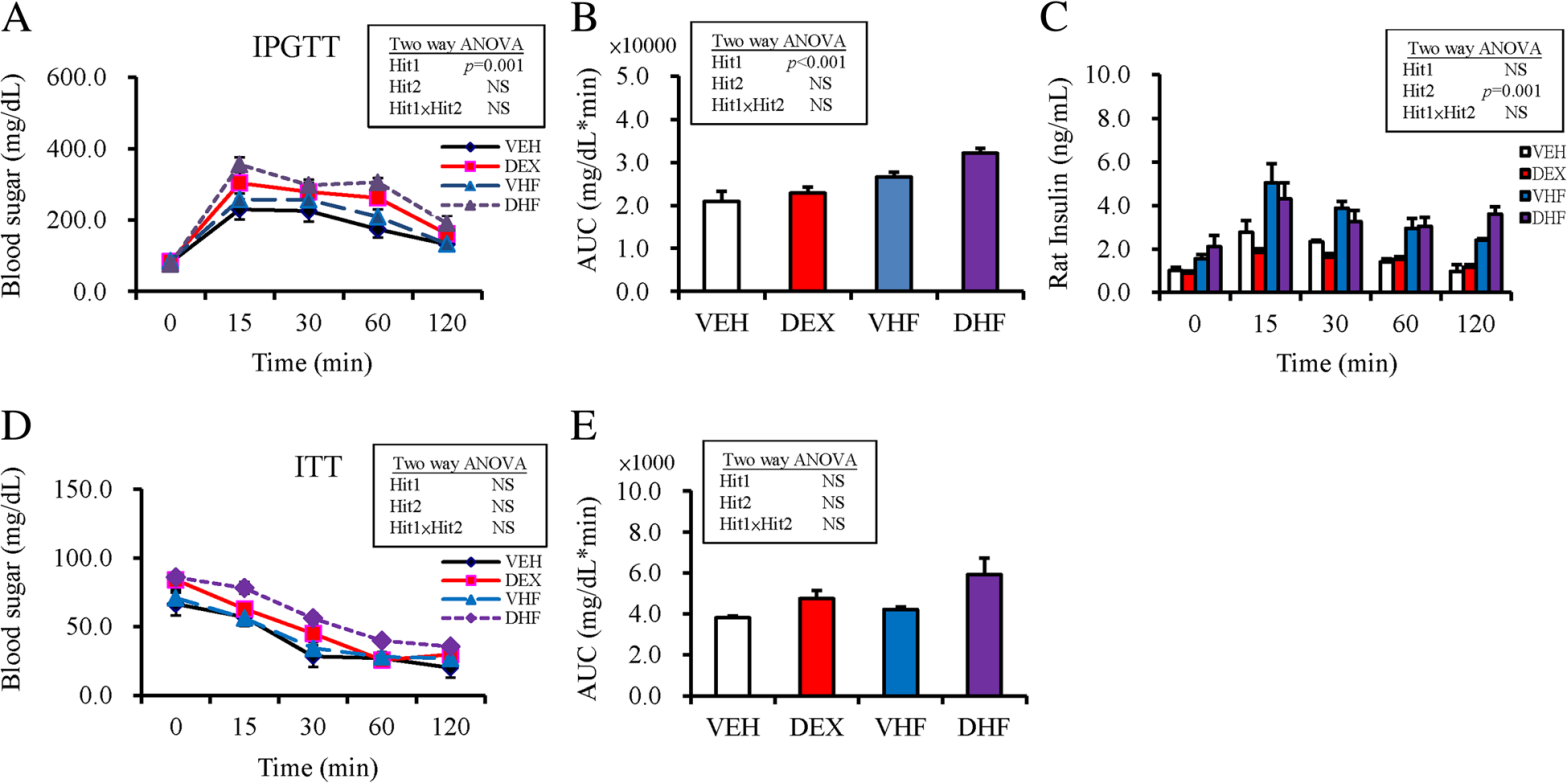
Blood glucose levels in post-stimulation tests. (**a**) Intraperitoneal glucose tolerance test (IPGTT); (**b**) Glucose area under the curve (AUC) of IPGTT; (**c**) plasma insulin level; (**d**) insulin tolerance test (ITT); and (**e**) AUC of ITT in the four experimental groups. Results were analyzed using two-way ANOVA (prenatal dexamethasone exposure × postnatal HF diet). All values are presented as the mean ± standard error (*n* = 8). *P < 0.05. VEH, normal diet; DEX, prenatal dexamethasone exposure; VHF, postnatal HF diet; DHF, prenatal dexamethasone plus postnatal HF diet

The results of intraperitoneal glucose tolerance test showed that prenatal dexamethasone treatment showed a significant effect, whereas postnatal HF diet showed no effect on glucose AUC; there was no interaction between the treatments (Fig. 1a, b). Using two-way ANOVA, we showed that postnatal HF diet exerted significant effect (Hit 2, *p* < 0.001), whereas postnatal dexamethasone exposure (Hit 1) exhibited no significant effect on plasma insulin level; there was no interaction between treatments (Fig. 1c). The results of the ITT test showed that prenatal DEX treatment (Hit 1) and postnatal HF diet (Hit 2) showed no effect on blood sugar and there was no interaction between treatments (Fig. 1d). The treatments showed similar results on insulin AUC (Fig. 1e).

### Prenatal dexamethasone and postnatal HF diet induce chronic inflammation and leptin dysregulation

**Fig. 2:**
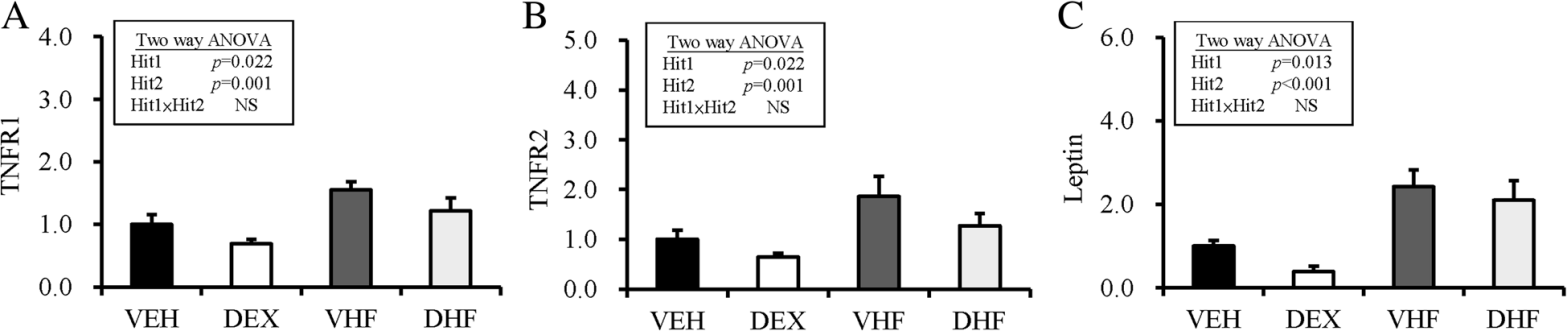
Hit 1/Hit 2 (H1/H2) induced chronic inflammation and leptin dysregulation in visceral adipose tissue. Fold change in the mRNA expression of (**a**) TNF-receptor 1 (TNF-R1), (**b**) TNF-R2, and (**c**) leptin in retroperitoneal adipose tissue was decreased by prenatal dexamethasone exposure and increased by postnatal high-fat (HF) diet. Results were analyzed using two-way ANOVA (prenatal dexamethasone exposure × postnatal HF diet). All values are presented as the mean ± standard error (*n* = 8). *P < 0.05. VEH, normal diet; DEX, prenatal dexamethasone exposure; VHF, postnatal HF diet; DHF, prenatal dexamethasone plus postnatal HF diet

Animals in the DHF group had higher AST and ALT levels than those in the other three groups, but the difference was not statistically significant (Table 1). Using two-way ANOVA, we showed that prenatal dexamethasone treatment (Hit 1) showed no effect, whereas postnatal HF diet had a positive effect on AST and ALT levels (Hit 2, *p* < 0.001); however, the effect was not significant and there was no interactive effect. Using two-way ANOVA we also showed that prenatal dexamethasone treatment exerted no significant effect, whereas postnatal HF diet had positive effects (*p* < 0001) on plasma leptin, TNF-α, and VEGF levels (Table 1). The DHF group showed higher level of plasma TNF-α than those of the other groups (Table 1). We also determined the mRNA expression of TNF-receptors in retroperitoneal adipose tissue (Fig. 2). The mRNA expression of TNF-receptor 1 (TNF-R1) and TNF-R2 in retroperitoneal adipose tissue was decreased by prenatal dexamethasone exposure and increased by postnatal HF diet, but there was no interaction between the two treatments (Fig. 2a, b). The mRNA expression of leptin in retroperitoneal adipose tissue was also decreased by prenatal dexamethasone exposure (Hit 1, *p* = 0.013) and increased by postnatal HF diet (Hit 2, *p* = 0.001) without interaction between treatments.

### Prenatal dexamethasone and postnatal HF diet exhibit distinct influences on the nutrient-sensing pathway in retroperitoneal adipose tissue

Nutrient-sensing signals in retroperitoneal adipose tissue were observed to investigate the programming effects of prenatal dexamethasone treatment and postnatal HF diet.

**Fig. 3:**
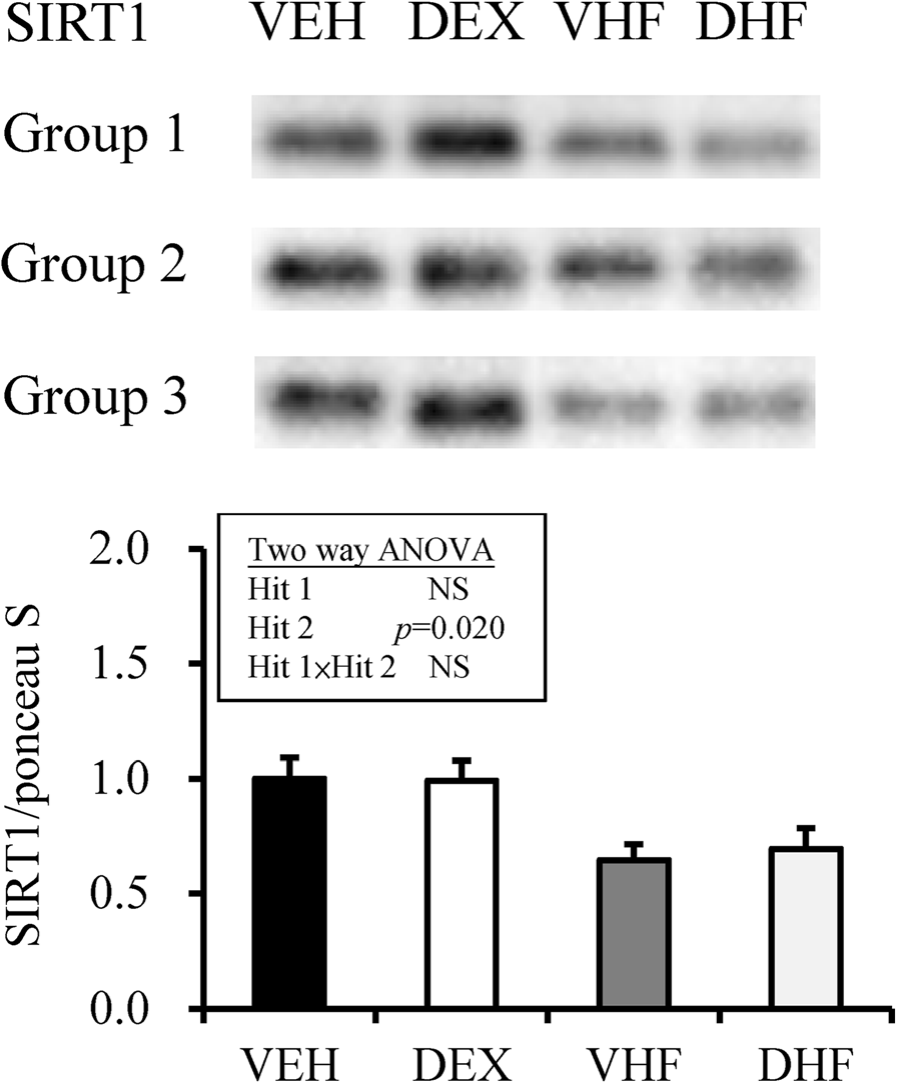
Results of western blotting analysis of SIRT1 in the four experimental groups. SIRT1 abundance is shown after normalization with ponceau S abundance. The abundance of SIRT1 protein was decreased by postnatal high-fat (HF) diet exposure. VEH, normal diet; DEX, prenatal dexamethasone exposure; VHF, postnatal HF diet; DHF, prenatal dexamethasone plus postnatal HF diet

**Fig. 4:**
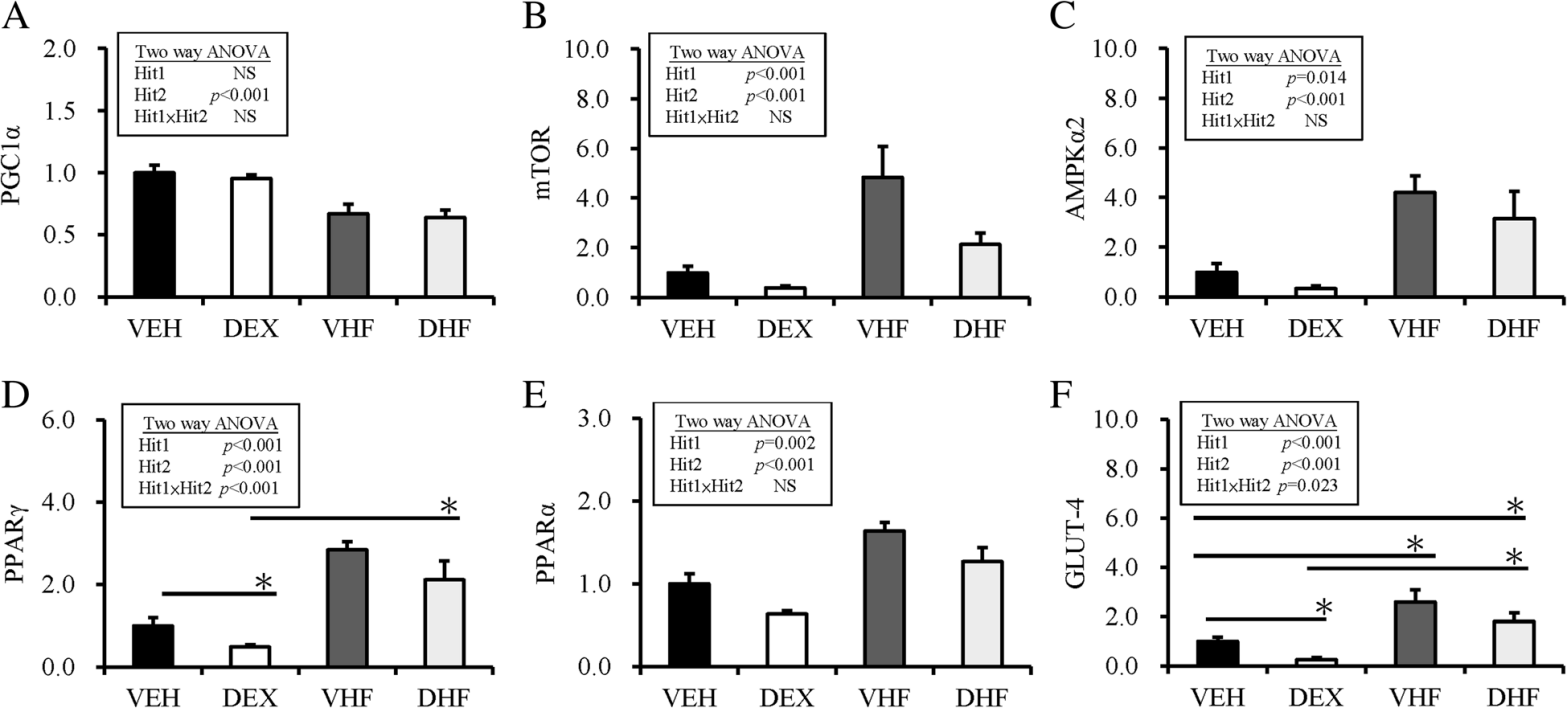
Hit 1/Hit 2 (H1/H2) caused alteration in nutrition sensory signaling. Fold change in the mRNA expression of nutrition sensory signals in retroperitoneal adipose tissue as determined by qRT-PCR: (**a**) PGC1-α, (**b**) mTOR, (**c**) AMPK-α2, (**d**) PPAR-γ, (**e**) PPAR-α, and (**f**) GLUT4. Results were analyzed using two-way ANOVA (prenatal dexamethasone exposure × postnatal HF diet). All values are presented as the mean ± standard error (*n* = 8). *P < 0.05. VEH, normal diet; DEX, prenatal dexamethasone exposure; VHF, postnatal HF diet; DHF, prenatal dexamethasone plus postnatal HF diet

Sirtuin 1 (SIRT1) is an important regulator of energy homeostasis in response to nutrient availability. It regulates lipid homeostasis and insulin resistance by regulating peroxisome proliferators-activated receptor α (PPARα)/PGC1-α and GLUT4, respectively [20, 21]. Results of western blotting analysis revealed that the abundance of SIRT1 protein was decreased by postnatal HF diet exposure (Fig. 3). For rapid dynamic change of nutrient-sensing signal, the mRNA expression of other nutrient-sensing molecules was investigated. As showed in Fig. 4, by two-way ANOVA, we revealed that the mRNA expression of PGC1-α in retroperitoneal adipose tissue was decreased by postnatal HF diet (Hit 2, *p* < 0.001), but not by prenatal dexamethasone treatment (Hit 1), and there was no interaction between Hit 1 and Hit 2. The mRNA expression of mTOR, AMPK-α2, and PPAR-α in retroperitoneal adipose tissue was suppressed by prenatal dexamethasone treatment (*p* < 0.001) but enhanced by postnatal HF diet (*p* < 0.001), without significant interaction between Hit 1 and Hit 2. The mRNA expression of PPAR-γ and GLUT4 was also decreased by prenatal dexamethasone exposure but increased by postnatal HF diet, and there was a significant interaction between Hit 1 and Hit 2. Although prenatal dexamethasone treatment and postnatal HF diet had synergistic effect on BW and retroperitoneal adiposity, they displayed distinct modulating effects on the nutrient-sensing signals of retroperitoneal adipose tissue (Table 2).

**Table 2:** Nutrition sensory signal pathway alteration by prenatal dexamethasone and postnatal high-fat diet treatment

Nutrition sensory molecule	Prenatal Dexamethasone	Postnatal high-fat diet	h
SIRT1	→	↓	X
PGC1α	→	↓	X
mTOR	↓	↑	X
AMPK-α2	↓	↑	X
PPAR-α	↓	↑	X
PPAR-γ	↓	↑	O
GLUT4	↓	↑	O

→ no change, ↑ increase, ↓ decrease

*X* no interaction, *O* interaction

### Prenatal dexamethasone and postnatal HF diet induce desynchronization of the expression of circadian clock genes

**Fig. 5:**
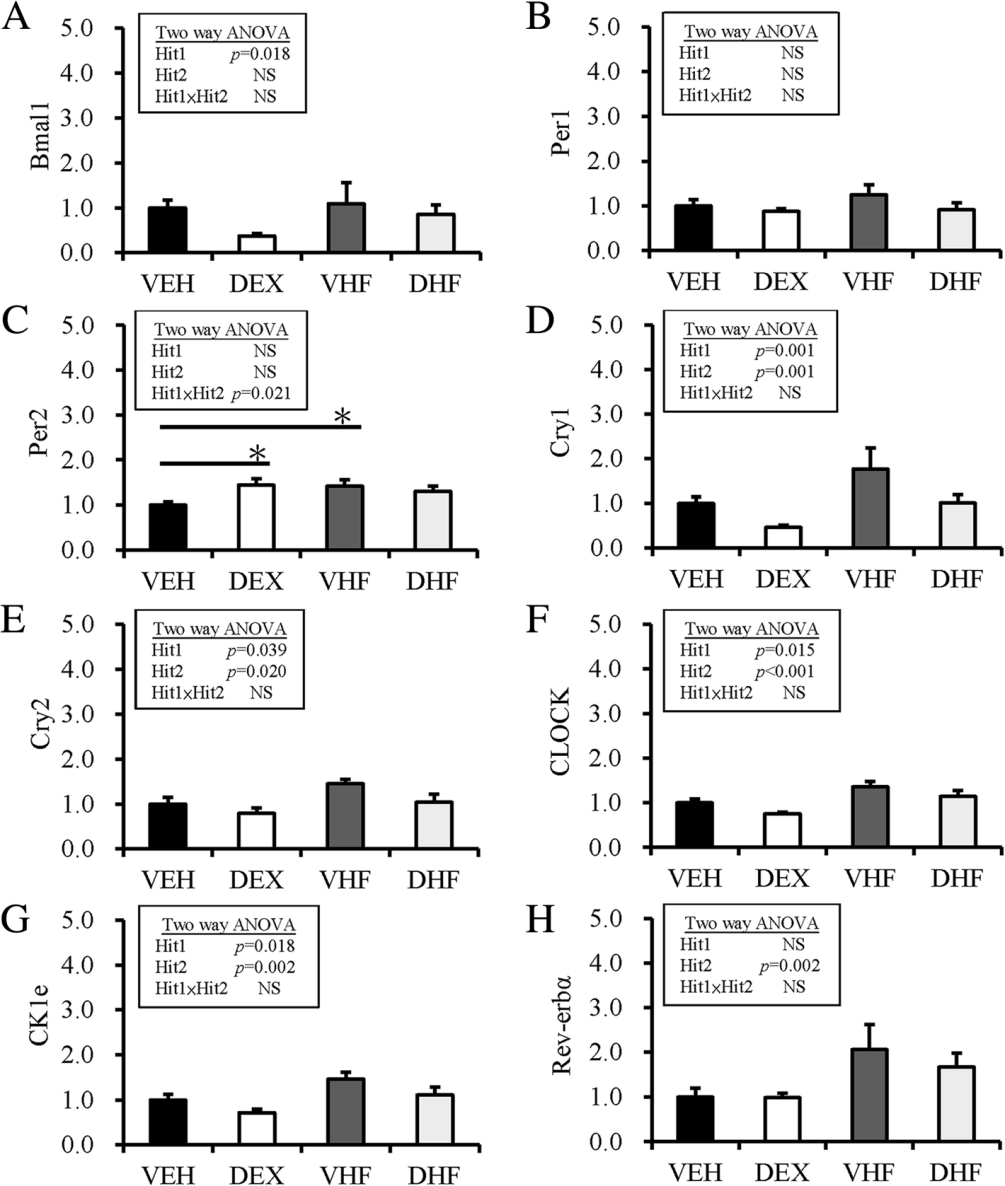
H1/H2 altered the expression of circadian-clock genes in visceral adipose tissue. The effects of prenatal dexamethasone and postnatal HF diet on the expression of the circadian-clock genes (**a**) Bmal-1, (**b**) Per1, (**c**) Per2, (**d**) Cry1, (**e**) Cry2, (**f**) CLOCK, (**g**) Ck1e, and (**h**) Rev-erbα. Results were analyzed using two-way ANOVA (prenatal dexamethasone exposure × postnatal HF diet). All values are presented as the mean ± standard error (*n* = 8). *P < 0.05. VEH, normal diet; DEX, prenatal dexamethasone exposure; VHF; postnatal HF diet; DHF, prenatal dexamethasone plus postnatal HF diet

Recent studies have revealed the relationship between desynchronization of clock genes in adipose tissue and development of obesity [22, 23]. Thus, the effects of prenatal dexamethasone and postnatal HF diet on the mRNA expression of circadian rhythm-associated genes were further tested. The mRNA expression of Bmal-1 was affected by prenatal DEX treatment but not by postnatal HF diet (Fig. 5a). Both Hit 1 and Hit 2 did not significantly affect the mRNA expression of Per1 and Per2 in retroperitoneal adipose tissue (Fig. 5b and c). Prenatal dexamethasone treatment decreased the mRNA expression of Cry1 and Cry2, whereas postnatal HF diet increased the mRNA expression of Cry1 and Cry2 in retroperitoneal adipose tissue without interaction between the two treatments (Fig. 5d and e). Similarly, the mRNA expression of CLOCK and CKle was decreased by prenatal dexamethasone treatment but increased by postnatal HF diet without significant interaction between Hit 1 and Hit 2 (Fig. 5f and g). The mRNA expression of Rev-erbα in retroperitoneal adipose tissue was enhanced by postnatal HF diet (Hit 2, *p* = 0.002) but not by prenatal DEX treatment, and without significant interaction between Hit 1 and Hit 2 (Fig. 5h).

## Discussion

In this present study, we used an animal model to investigate the effects of prenatal dexamethasone (Hit 1) and postnatal HF diet (Hit 2) on metabolic manifestations. Prenatal dexamethasone treatment was used as a prenatal stress model because fetus are often over-exposed to glucocorticoid during prenatal stress [24, 25]. Prenatal dexamethasone treatment has been shown to enhance the susceptibility of offspring to postnatal HF diet-induced programmed obesity, insulin dysregulation, and hypertension [6, 9, 10]. Here, we further addressed the dysregulation of nutrient-sensing molecules and circadian-clock genes in retroperitoneal adipose tissue induced by prenatal dexamethasone and postnatal HF treatments. Our study showed that prenatal dexamethasone plus postnatal HF diet exhibited synergistic effects on body and visceral adiposity. Moreover, prenatal dexamethasone treatment and postnatal HF diet showed distinct influences on nutrient-sensing molecules in retroperitoneal adipose tissue. Prenatal dexamethasone treatment and postnatal HF diet also altered the expression of circadian-clock genes in retroperitoneal adipose tissue.

Chronic inflammation and adipokine dysregulation are thought to be characteristics of obesity. It has been reported that TNFα concentration in adipose tissue is correlated with obesity and insulin resistance in patients with and without type 2 diabetes [26, 27]. TNFα may increase systemic insulin resistance by promoting the release of fatty acids from adipose tissue into the bloodstream to act on tissues, such as muscle and liver tissues. Thus, TNFα can act locally in adipose tissue, which ultimately promotes insulin resistance in peripheral tissues [28]. In our study, we found that plasma TNFα level was increased by postnatal HF exposure but not by prenatal dexamethasone treatment. Although prenatal dexamethasone treatment appeared not to increase plasma TNFα level, it decreased the mRNA expression of TNF-R1 and TNF-R2 in retroperitoneal adipose tissue. This result suggested that prenatal dexamethasone treatment may lead to TNFα resistance through inhibition of TNF-Rs in local adipose tissue.

SIRT1, a highly conserved NAD^+^-dependent protein deacetylase, has been recognized as a key metabolic sensor that directly links environmental nutrient signals to metabolic homeostasis [29]. SIRT1 has multiple important functions, such as decrease adipokine secretion, anti-inflammation, maintain glucose and lipid homeostasis, decrease oxidative stress, regulate mitochondrial function, and circadian rhythms. SIRT1 can repress the translocation of PPARγ and inhibit lipid accumulation in adipose tissue [20]. In well differentiated adipocytes, SIRT1 is upregulated, thereby triggering lipolysis and loss of fat [30]. SIRT1 is also a functional regulator of PPARα and its coactivator PGC-1α, which induce a metabolic transcription program of mitochondrial fatty acid oxidation and lipid homeostasis [20, 31]. Thus, decreased SIRT-1/PGC1α and increased PPARγ/PPARα in retroperitoneal adipose tissue correspond to the dysregulation of lipid homeostasis associated with postnatal HF diet treatment. In contrast, decreased PPARγ and PPARα in the retroperitoneal adipose tissue of rats exposed to prenatal dexamethasone suggested that prenatal dexamethasone exposure programmed adiposity using different mechanisms. SIRT1 is also involved in glucose metabolism by increasing insulin secretion by pancreatic β-cells and modulation of insulin signaling [32]. The activation of SIRT1 can improve insulin resistance, increase fatty acid oxidation, and mitochondrial biogenesis in the skeletal muscle. In addition, PGC-1α can upregulate glucose transporter 4 (GLUT4) expression and glucose transport activity [33]. AMPK has been described to directly affect SIRT-1/PGC-1α activity through phosphorylation and deacetylation, respectively [34]. It was revealed that SIRT1 exerts a regulatory role in glucose metabolism through PGC-1α/GLUT4 modulation. Alterations in these indicated signal molecules suggest that we need a different therapeutic strategy to treat metabolic dysregulations programmed by prenatal dexamethasone and postnatal HF diet treatment.

Mammals have circadian rhythms involving a set of circadian-clock genes, in order to adapt to environmental oscillations. In addition to circadian rhythms, these circadian-clock genes also play important roles in energy homeostasis and metabolism [32]. Turek et al. illustrated that homozygous *Clock* mutant mice can develop a metabolic syndrome showing hyperglycemia, hypoinsulinemia, and hepatic steatosis [23]. The circadian-clock is regulated by negative-feedback loops mediated by the heterodimeric transcription factors CLOCK-BMAL1 and their transcriptional targets, including PER and CRY proteins that directly repress CLOCK-BMAL1 activity, as well as REV-ERB and ROR nuclear receptors that control BMAL1 expression [35]. In our study, we analyzed the mRNA expression of genes associated with circadian-clock, and showed that the mRNA expression of Bmal-1, cry1, cry2, Clock, and CKle was affected by prenatal dexamethasone treatment. The mRNA expression of cry1, cry2, Clock, CKle1, and Rev-erbα were affected by postnatal HF diet. Thus, prenatal dexamethasone treatment and postnatal HF showed different influences on the expression of circadian-clock genes.

The reciprocal relationship between nutrient sensing and circadian clock had addressed [14–16]. For example, AMPK can trigger the phosphorylation and degradation of PER/CRY of circadian-clock [36]. Circadian-clock can regulate nutrient sensing PPARαβγ and diet-induced obesity occur in CLOCK dominant negative mutant mice [14]. NAD^+^/SIRT-1 are suggested as integrators of circadian rhythms and nutrient sensing pathway [14]. Studies have shown that SIRT1 interacts with CLOCK-BMAL1 to directly regulate the expression of circadian clock-controlled genes through deacetylation of PER2 and/or BMAL1 [37, 38]. In contrast, the NAD^+^ salvage also can be regulated by CLOCK-BMAL1 [39]. These findings highlight the interdependence of nutrient sensing and circadian clock.

## Conclusion

In summary, our study showed that obesity programmed by prenatal dexamethasone and postnatal HF diet lead to distinct alterations in nutrition sensory signals and circadian-clock genes in retroperitoneal adipose tissue. Consequently, the representation of resetting nutrient-sensing molecules and circadian-clock genes has potential therapeutic connection in regard to the pathogenesis and treatment of prenatal stress and postnatal HF diet-related metabolic disorders.

## Abbreviations

ALT: alanine transaminase
AST: aspartate transaminase
BW: Body weight
DEX: Prenatal dexamethasone exposure
DHF: prenatal dexamethasone exposure with postnatal HF diet
GC: Glucocorticoid
HDL: High-density lipoprotein
HF: Postnatal high-fat
PPARα: Peroxisome proliferators-activated receptor α
qRT-PCR: Quantitative reverse transcription-polymerase chain reaction
SD: Sprague-Dawley rats
SIRT1: Sirtuin 1
TNF-: TNF-receptor 1
TNF-α: Tumor necrosis factor-α
VEGF: Vascular endothelial growth factor
VEH: Vehicle group
VHF: Postnatal HF diet
